# Supplementary material for: Missense variant analysis in the TRPV1 ARD reveals the unexpected functional significance of a methionine
Source: PLoS One. 2025 Sep 2;20(9):e0331224. doi: 10.1371/journal.pone.0331224 (PMC12404443; doi:10.1371/journal.pone.0331224)
Supplement: S1 Fig — Fractional SASA score (pdb structure file: 7lp9) is plotted against SASA ranked amino acids of the ARD (residues 111–361) in rat TRPV1 (purple). Missense variant type count in gnomAD 4.1 for each position (black marker) is indicated on the right-side y-axis. SASA ranked amino acid X = 35 corresponds to M308 in the rat TRPV1 sequence and as indicated on the top left box. The Matlab script for generating this plot is described in Mott et al. (2023) and available through GitHub (esenlab/variant-analysis). (PDF) [file pone.0331224.s001.pdf]

Supporting information Figure 1

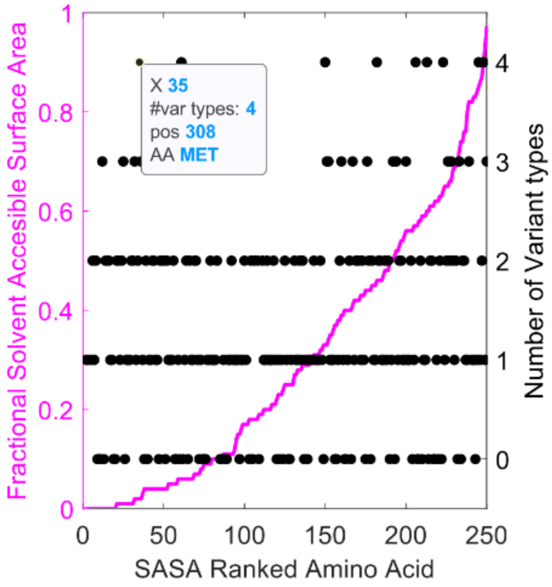

Figure S1. Variant number versus ranked SASA score. Fractional SASA score (pdb structure file: 7lp9) is plotted against SASA ranked amino acids of the ARD (residues 111-361) in rat TRPV1 (purple). Missense variant type count in gnomAD 4.1 for each position (black marker) is indicated on the right-side y-axis. SASA ranked amino acid X=35 corresponds to M308 in the rat TRPV1 sequence and as indicated on the top left box. The Matlab script for generating this plot is described in Mott et al. (2023) and available through gGitHub (*esenlab*/variant-analysis).
