# Supplementary material for: Missense variant analysis in the TRPV1 ARD reveals the unexpected functional significance of a methionine
Source: PLoS One. 2025 Sep 2;20(9):e0331224. doi: 10.1371/journal.pone.0331224 (PMC12404443; doi:10.1371/journal.pone.0331224)
Supplement: S2 Fig — (A) Macroscopic current voltage clamp experiment with application of capsaicin and MTSEA as indicated over traces. Control TRPV1-C157A with 1 mM MTSEA and co-application of 0.3 μM capsaicin. (B) TRPV1-C157A/M308C with 1 mM MTSEA and co-application of 0.3 μM capsaicin. (PDF) [file pone.0331224.s002.pdf]

Supporting information Figure 2

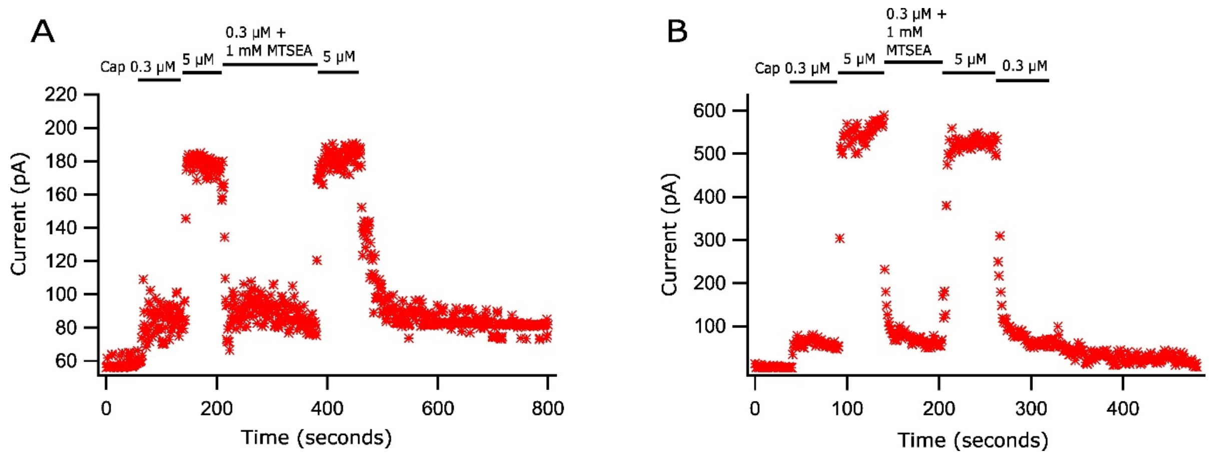

Figure S2. Application of MTSEA to TRPV1-M308C/C157A excised patches does not affect channel activity. (A) Macroscopic current voltage clamp experiment with application of capsaicin and MTSEA as indicated over traces. Control TRPV1-C157A with 1 mM MTSEA and co-application of 0.3 µM capsaicin. **(B)** TRPV1-C157A/M308C with 1 mM MTSEA and co-application of 0.3 µM capsaicin.
