# Supplementary material for: Missense variant analysis in the TRPV1 ARD reveals the unexpected functional significance of a methionine
Source: PLoS One. 2025 Sep 2;20(9):e0331224. doi: 10.1371/journal.pone.0331224 (PMC12404443; doi:10.1371/journal.pone.0331224)
Supplement: S4 Fig — (A) Currents collected in voltage sweeps with voltage set to +80mV. Because the voltage sweeps include a -80mV and +80mV segment, we used a macro to extract the + 80mV segments and concatenate these in the presented and analyzed data (described in the Methods section). Current traces with single and double channel contributions are colored, and the multi-channel patch is black. Notable properties of the green trace is high activity for two channels in the wash buffer before and after capsaicin application. (B) Summary plot of the channel activity illustrated in data traces shown in (A). The multi-channel patch is plotted with a line and single channel data is plotted with colored markers. The purple trace shows two active channels until the application of the 5 μM solution when one channel becomes silent. The purple 5μM concentration data point in (B) is based on a single channel remaining active in the purple trace. (PDF) [file pone.0331224.s004.pdf]

Supporting information Figure 4

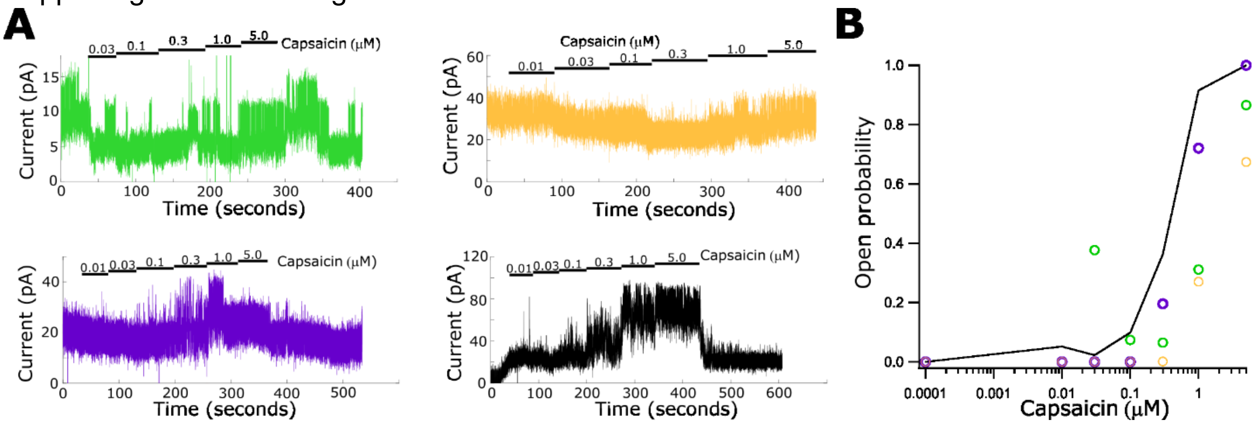

Figure S4. TRPV1M308H dose response data with single channel and multi-channel patches. (A) Currents collected in voltage sweeps with voltage set to +80mV. Because the voltage sweeps include a -80mV and +80mV segment, we used a macro to extract the +80mV segments and concatenate these in the presented and analyzed data (described in the Methods section). Current traces with single and double channel contributions are colored, and the multi-channel patch is black. Notable properties of the green trace is high activity for two channels in the wash buffer before and after capsaicin application. **(B)** Summary plot of the channel activity illustrated in data traces shown in (A). The multi-channel patch is plotted with a line and single channel data is plotted with colored markers. The purple trace shows two active channels until the application of the 5 μM solution when one channel becomes silent. The purple 5μM concentration data point in (B) is based on a single channel remaining active in the purple trace.
