# Supplementary material for: Missense variant analysis in the TRPV1 ARD reveals the unexpected functional significance of a methionine
Source: PLoS One. 2025 Sep 2;20(9):e0331224. doi: 10.1371/journal.pone.0331224 (PMC12404443; doi:10.1371/journal.pone.0331224)
Supplement: S5 Fig — (A) Inside out patch with 5 WT channels from an experiment also included in wild type sample from Fig 3D. (B) Inside out patch with 5 TRPV1-M308H channels with similar current response profile to single channel experiments shown in Figure 3. (C) Summary data of normalized responses in experiments from panels A and B. (PDF) [file pone.0331224.s005.pdf]

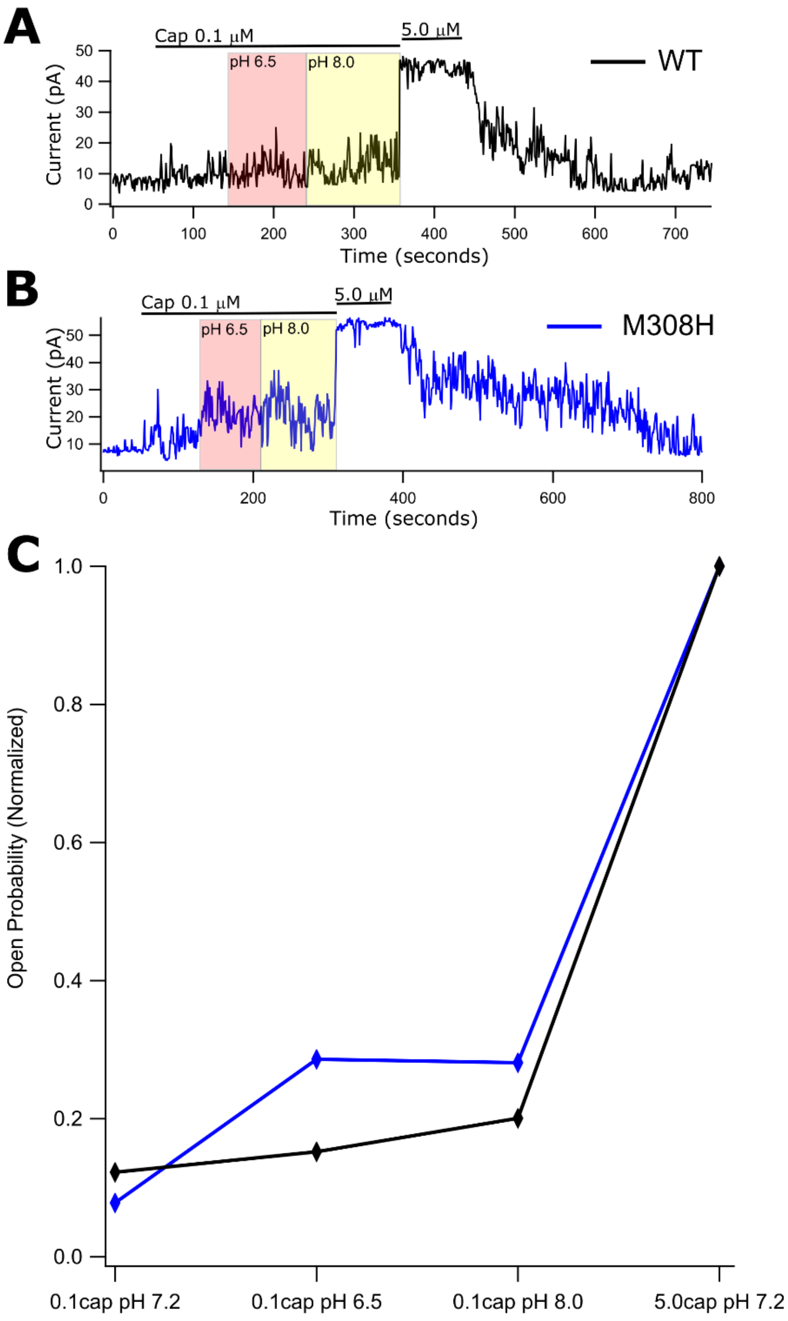

Figure S5. Modulation of currents by pH on inner leaflet of excised membrane patch from cells expressing reduced wild type TRPV1 and TRPV1-M308H. (A) Inside out patch with 5 WT channels from an experiment also included in wild type sample from Figure 3D. (B) Inside out patch with 5 TRPV1-M308H channels with similar current response profile to single channel experiments shown in Figure 3. (C) Summary data of normalized responses in experiments from panels A and B.
