# Supplementary material for: Missense variant analysis in the TRPV1 ARD reveals the unexpected functional significance of a methionine
Source: PLoS One. 2025 Sep 2;20(9):e0331224. doi: 10.1371/journal.pone.0331224 (PMC12404443; doi:10.1371/journal.pone.0331224)
Supplement: S1 Table — Excerpt of Alphamissense scores assigned to M309 in human TRPV1 (M308 in the rat TRPV1 sequence). SNV, indicates with “y” whether a single nucleotide variant is known to exist. (PDF) [file pone.0331224.s006.pdf]

Supporting information Tables

Supporting information Table 1

Uniprot ID	Entry name	Ensemble ID	Protein variant	SNV	AM Path. Score	Pathogenicity class
Q8NER1	TRPV1_HUMAN	ENST00000399756.8	p.Met309Ala		0.8691	likely_pathogenic
Q8NER1	TRPV1_HUMAN	ENST00000399756.8	p.Met309Cys		0.9186	likely_pathogenic
Q8NER1	TRPV1_HUMAN	ENST00000399756.8	p.Met309Asp		0.9988	likely_pathogenic
Q8NER1	TRPV1_HUMAN	ENST00000399756.8	p.Met309Glu		0.9833	likely_pathogenic
Q8NER1	TRPV1_HUMAN	ENST00000399756.8	p.Met309Phe		0.901	likely_pathogenic
Q8NER1	TRPV1_HUMAN	ENST00000399756.8	p.Met309Gly		0.9855	likely_pathogenic
Q8NER1	TRPV1_HUMAN	ENST00000399756.8	p.Met309His		0.9861	likely_pathogenic
Q8NER1	TRPV1_HUMAN	ENST00000399756.8	p.Met309Ile	y	0.7738	likely_pathogenic
Q8NER1	TRPV1_HUMAN	ENST00000399756.8	p.Met309Lys	y	0.9446	likely_pathogenic
Q8NER1	TRPV1_HUMAN	ENST00000399756.8	p.Met309Leu	y	0.5779	likely_pathogenic
Q8NER1	TRPV1_HUMAN	ENST00000399756.8	p.Met309Asn		0.9753	likely_pathogenic
Q8NER1	TRPV1_HUMAN	ENST00000399756.8	p.Met309Pro		0.9981	likely_pathogenic
Q8NER1	TRPV1_HUMAN	ENST00000399756.8	p.Met309Gln		0.8607	likely_pathogenic
Q8NER1	TRPV1_HUMAN	ENST00000399756.8	p.Met309Arg	y	0.933	likely_pathogenic
Q8NER1	TRPV1_HUMAN	ENST00000399756.8	p.Met309Ser		0.9081	likely_pathogenic
Q8NER1	TRPV1_HUMAN	ENST00000399756.8	p.Met309Thr	y	0.6428	likely_pathogenic
Q8NER1	TRPV1_HUMAN	ENST00000399756.8	p.Met309Val	y	0.1557	likely_benign
Q8NER1	TRPV1_HUMAN	ENST00000399756.8	p.Met309Trp		0.9922	likely_pathogenic
Q8NER1	TRPV1_HUMAN	ENST00000399756.8	p.Met309Tyr		0.9875	likely_pathogenic

Table S1. Alphamissense pathogenicity of M309 position in TRPV1. Excerpt of Alphamissense scores assigned to M309 in human TRPV1 (M308 in the rat TRPV1 sequence). SNV, indicates with “y” whether a single nucleotide variant is known to exist.
