# Supplementary material for: Missense variant analysis in the TRPV1 ARD reveals the unexpected functional significance of a methionine
Source: PLoS One. 2025 Sep 2;20(9):e0331224. doi: 10.1371/journal.pone.0331224 (PMC12404443; doi:10.1371/journal.pone.0331224)
Supplement: Attachment — Submitted filename: Review_PlosONE.pdf [file pone.0331224.s009.pdf]

The general quality of the data presented in the manuscript is good, and the study addresses a relevant scientific question. However, the presentation of the data lacks clarity and, at times, appears sloppy. This significantly affects the readability and interpretation of the results. I have several major comments that need to be addressed to improve the manuscript.

1) Methods – Molecular Biology
"In vitro assembly (IVA)" – According to the original paper describing the method (https://doi.org/10.1038/srep27459), IVA stands for ***In Vivo Assembly***, not ***In Vitro Assembly***.

2) Results – "Capsaicin sensitivity of M308 mutants points to side chain constraints in TRPV1 ARD"
Please describe the functional effects observed with the M308I substitution in more detail.

3) Protonation of M308H modulates TRPV1 function
"The size-dependence of side-chain substitutions at M308, as well as a gain-of-function phenotype observed when ***Ile is substituted for Met or Val,*** led us to hypothesize that covalent modification of the side-chain could result in channel activation or sensitization."

Did you mean substitution of Met to Ile or Val? Please clarify the direction of the mutation.

I also find the conclusions drawn from these experiments problematic. The data suggest that M308H reduces expression or trafficking of functional channels to the membrane. This could potentially be tested experimentally, but as it stands, the mutation appears detrimental.
Additionally, I am not an expert in single-channel recordings and analysis, but the data presentation is not sufficiently clear. It would be helpful to include wild-type TRPV1 single-channel recordings and a comparison of open probability values.

4) "A physiologically relevant role for this shorter splice variant could provide an explanation for why the methionine at position 308 is so highly conserved, ***even though other residues at that position result in channels with behavior that is similar to WT."***

This is a controversial statement. You only demonstrated similar behavior for Ala, Val, and slightly changed for Ile. The substitutions to Trp rendered the channel inactive. The His substitution significantly reduced current amplitude.

5) Methods – Cell response counts from truncated TRPV1 co-expressed with TRPV1 wild type
"Truncated constructs (TRPV1-preDelM308 and TRPV1-5'sv) were co-expressed with TRPV1 wild-type constructs at a 25% to 75% plasmid DNA weight ratio: TRPV1 wild type (25%) and PLC1-PH-SNAPtag (75%, a plasmid membrane marker for PIP2); TRPV1 wild type (25%) and TRPV1-preDelM308 (75%); TRPV1 wild type (25%) and TRPV1-5'sv (75%)."

It might be informative to try additional plasmid DNA ratios (e.g., 50:50 or 75:25). This could help clarify how dominant the effect is. How many subunits of the truncated variant are needed to prevent trafficking of the full channel to the membrane?

Minor comments
6) Figure 1D
Correct "Argenin" to Arginine.
I recommend using the format MXXX rather than writing "Met in TRPA1 ARD" for consistency and clarity.
Consider using EC_{50} instead of $K_1/_2$ to improve accessibility for a broader journal audience.

7) Figure 2C
Use °C instead of "deg C".
Also, use “WT” instead of “Wild type TRPV1” for consistency with other figures.

8) Figure 3B
In the zoomed-in regions of the current recordings (i.–iii.), the time markers do not appear to match those in the main figure. Please double-check.
Figure 3D
Use either Caps or Cap consistently throughout the figure and text when referring to capsaicin
