# Supplementary material for: Missense variant analysis in the TRPV1 ARD reveals the unexpected functional significance of a methionine
Source: PLoS One. 2025 Sep 2;20(9):e0331224. doi: 10.1371/journal.pone.0331224 (PMC12404443; doi:10.1371/journal.pone.0331224)
Supplement: Attachment — Submitted filename: Response to Reviewers - GWulffraatEtAl_PLOSOne.docx [file pone.0331224.s011.docx]

Dear editors and reviewers,

We thank the editorial staff and reviewers for their efforts to improve the manuscript, “Missense variant analysis in the TRPV1 ARD reveals the unexpected functional significance of a methionine”. We have given consideration to both the stylistic criteria of the journal and reviewers’ suggested revisions to our manuscript and believe that many of the recommendations could be accommodated. One of the key requirements made by the editors was providing access to the data underlying our analyses and figures. We have made these available now as spreadsheets in “Supplemental_Data”, providing the associated figure in the file name.

The manuscript edits required by the journal were made without “track changes” while the alterations made at the behest of reviewers were made with “track changes” active. This will facilitate corroborating any of the changes indicated in our line-by-line responses below with the edits made to the manuscript. Lastly, one additional figure was added to the supplementary figures.

We have adopted the following changes to text so that our responses are easily noted:

Responses to reviewers are blue.

[*italicized text is added to manuscript*]

Sincerely,

Eric Senning

PONE-D-25-14924

Missense variant analysis in the TRPV1 ARD reveals the unexpected functional significance of a methionine.

PLOS ONE

Dear Dr. Senning,

Thank you for submitting your manuscript to PLOS ONE. The manuscript has been evaluated by two experts in the field. Both reviewers indicated that the manuscript reports interesting data. However, the reviewers also identified several problems. Thus, although the manuscript has merit, it does not fully meet PLOS ONE’s publication criteria as it currently stands. Therefore, we invite you to submit a revised version of the manuscript that addresses the concerns of the reviewers.

Please submit your revised manuscript by Jun 14 2025 11:59PM. If you will need more time than this to complete your revisions, please reply to this message or contact the journal office at [plosone@plos.org](mailto:plosone@plos.org). When you're ready to submit your revision, log on to <https://www.editorialmanager.com/pone/> and select the 'Submissions Needing Revision' folder to locate your manuscript file.

Please include the following items when submitting your revised manuscript:

- A rebuttal letter that responds to each point raised by the academic editor and reviewer(s). You should upload this letter as a separate file labeled 'Response to Reviewers'.
- A marked-up copy of your manuscript that highlights changes made to the original version. You should upload this as a separate file labeled 'Revised Manuscript with Track Changes'.
- An unmarked version of your revised paper without tracked changes. You should upload this as a separate file labeled 'Manuscript'.

If you would like to make changes to your financial disclosure, please include your updated statement in your cover letter. Guidelines for resubmitting your figure files are available below the reviewer comments at the end of this letter.

If applicable, we recommend that you deposit your laboratory protocols in protocols.io to enhance the reproducibility of your results. Protocols.io assigns your protocol its own identifier (DOI) so that it can be cited independently in the future. For instructions see: [https://journals.plos.org/plosone/s/submission-guidelines#loc-laboratory-protocols](https://nam12.safelinks.protection.outlook.com/?url=https%3A%2F%2Ftrack.editorialmanager.com%2FCL0%2Fhttps%3A%252F%252Fjournals.plos.org%252Fplosone%252Fs%252Fsubmission-guidelines%2523loc-laboratory-protocols%2F1%2F010f0196873c5369-494753d7-fe34-46c3-b215-91735291dfb5-000000%2FKOsyTOcIGvqoze1J4MBqjtozpWRXU2NivKFGcskt5n0%3D208&data=05%7C02%7C%7C45171c4bce9c4de2fccd08dd87f8e7a3%7C31d7e2a5bdd8414e9e97bea998ebdfe1%7C0%7C0%7C638816225327400503%7CUnknown%7CTWFpbGZsb3d8eyJFbXB0eU1hcGkiOnRydWUsIlYiOiIwLjAuMDAwMCIsIlAiOiJXaW4zMiIsIkFOIjoiTWFpbCIsIldUIjoyfQ%3D%3D%7C0%7C%7C%7C&sdata=WU25rRoKVgJYAScHjy83mU1gMJuvOhVvtXDpBLzPEJg%3D&reserved=0). Additionally, PLOS ONE offers an option for publishing peer-reviewed Lab Protocol articles, which describe protocols hosted on protocols.io. Read more information on sharing protocols at [https://plos.org/protocols?utm_medium=editorial-email&utm_source=authorletters&utm_campaign=protocols](https://nam12.safelinks.protection.outlook.com/?url=https%3A%2F%2Ftrack.editorialmanager.com%2FCL0%2Fhttps%3A%252F%252Fplos.org%252Fprotocols%253Futm_medium%3Deditorial-email%2526utm_source%3Dauthorletters%2526utm_campaign%3Dprotocols%2F1%2F010f0196873c5369-494753d7-fe34-46c3-b215-91735291dfb5-000000%2FYNFtZiNogP6awHoWUZAxT1jsHRNTHR-EjthUIUyzAsw%3D208&data=05%7C02%7C%7C45171c4bce9c4de2fccd08dd87f8e7a3%7C31d7e2a5bdd8414e9e97bea998ebdfe1%7C0%7C0%7C638816225327450833%7CUnknown%7CTWFpbGZsb3d8eyJFbXB0eU1hcGkiOnRydWUsIlYiOiIwLjAuMDAwMCIsIlAiOiJXaW4zMiIsIkFOIjoiTWFpbCIsIldUIjoyfQ%3D%3D%7C0%7C%7C%7C&sdata=lnD64tjphHuFRnSlqHi6ujRMyz3Q5yj7f0thJKyEJyg%3D&reserved=0).

We look forward to receiving your revised manuscript.

Kind regards,

Alexander G Obukhov, Ph.D.
Academic Editor
PLOS ONE

Journal Requirements:

When submitting your revision, we need you to address these additional requirements.

1. Please ensure that your manuscript meets PLOS ONE's style requirements, including those for file naming. The PLOS ONE style templates can be found at <https://journals.plos.org/plosone/s/file?id=wjVg/PLOSOne_formatting_sample_main_body.pdf> and <https://journals.plos.org/plosone/s/file?id=ba62/PLOSOne_formatting_sample_title_authors_affiliations.pdf>

We have modified the stylistic properties of the manuscript to match the style requirements of PLOS ONE.

2. We note that the grant information you provided in the ‘Funding Information’ and ‘Financial Disclosure’ sections do not match.

When you resubmit, please ensure that you provide the correct grant numbers for the awards you received for your study in the ‘Funding Information’ section.

 We have edited our acknowledgments section and removed the funding information from here so that it only appears in the Funding and financial disclosures section as described in your journal policies.

3. PLOS ONE now requires that authors provide the original uncropped and unadjusted images underlying all blot or gel results reported in a submission’s figures or Supporting Information files. This policy and the journal’s other requirements for blot/gel reporting and figure preparation are described in detail at <https://journals.plos.org/plosone/s/figures#loc-blot-and-gel-reporting-requirements> and <https://journals.plos.org/plosone/s/figures#loc-preparing-figures-from-image-files>. When you submit your revised manuscript, please ensure that your figures adhere fully to these guidelines and provide the original underlying images for all blot or gel data reported in your submission. See the following link for instructions on providing the original image data: <https://journals.plos.org/plosone/s/figures#loc-original-images-for-blots-and-gels>.
In your cover letter, please note whether your blot/gel image data are in Supporting Information or posted at a public data repository, provide the repository URL if relevant, and provide specific details as to which raw blot/gel images, if any, are not available. Email us at [plosone@plos.org](mailto:plosone@plos.org) if you have any questions.

  We have submitted no gel or blot results with this manuscript.

4. Thank you for stating the following in the Acknowledgments Section of your manuscript:
We thank Dr. Sharona Gordon for TRPV1.pcDNA3.1 and Dr. Jason McLellan for JSM-164 NW10 3C-GFP-HS (GFP). We also thank Dr. Shai Silberberg for a conversation that started the ball rolling on the mysterious M308 residue of TRPV1. This work was supported by start-up funds from the University of Texas at Austin (E.N.S.) and the NSF-MCB grant no. 2129209 (E.N.S.).

We note that you have provided funding information that is not currently declared in your Funding Statement. However, funding information should not appear in the Acknowledgments section or other areas of your manuscript. We will only publish funding information present in the Funding Statement section of the online submission form.
Please remove any funding-related text from the manuscript and let us know how you would like to update your Funding Statement. Currently, your Funding Statement reads as follows:
This work was supported by start-up funds from the University of Texas at Austin (E.N.S.) and the NSF-MCB grant no. 2129209 (E.N.S.).

Please include your amended statements within your cover letter; we will change the online submission form on your behalf.

  The current funding statement as described above is correct. We have modified the acknowledgements to remove any traces of support from this section.

5. We noted in your submission details that a portion of your manuscript may have been presented or published elsewhere. A previous version of this manuscript was uploaded to the Bioarxiv server. Please clarify whether this [conference proceeding or publication] was peer-reviewed and formally published. If this work was previously peer-reviewed and published, in the cover letter please provide the reason that this work does not constitute dual publication and should be included in the current manuscript.

  We submitted a version of this manuscript to bioArxiv as a pre-print. This version of the manuscript was not peer-reviewed. We address this in the cover letter.

6. We note that you have indicated that there are restrictions to data sharing for this study. PLOS only allows data to be available upon request if there are legal or ethical restrictions on sharing data publicly. For more information on unacceptable data access restrictions, please see <http://journals.plos.org/plosone/s/data-availability#loc-unacceptable-data-access-restrictions>.

Before we proceed with your manuscript, please address the following prompts:

a) If there are ethical or legal restrictions on sharing a de-identified data set, please explain them in detail (e.g., data contain potentially identifying or sensitive patient information, data are owned by a third-party organization, etc.) and who has imposed them (e.g., a Research Ethics Committee or Institutional Review Board, etc.). Please also provide contact information for a data access committee, ethics committee, or other institutional body to which data requests may be sent.

b) If there are no restrictions, please upload the minimal anonymized data set necessary to replicate your study findings to a stable, public repository and provide us with the relevant URLs, DOIs, or accession numbers. For a list of recommended repositories, please see
<https://journals.plos.org/plosone/s/recommended-repositories>. You also have the option of uploading the data as Supporting Information files, but we would recommend depositing data directly to a data repository if possible.

We will update your Data Availability statement on your behalf to reflect the information you provide.

 We will comply with the expectation that the data is made available in a folder of excel spreadsheets. The spreadsheets are organized in folders pertaining to each of the figures in the manuscript.

7. In the online submission form, you indicated that our data include larger sets of images acquired in calcium imaging movies. We therefore will provide reasonable access to these for a specific range of images upon request.

All PLOS journals now require all data underlying the findings described in their manuscript to be freely available to other researchers, either 1. In a public repository, 2. Within the manuscript itself, or 3. Uploaded as supplementary information.
This policy applies to all data except where public deposition would breach compliance with the protocol approved by your research ethics board. If your data cannot be made publicly available for ethical or legal reasons (e.g., public availability would compromise patient privacy), please explain your reasons on resubmission and your exemption request will be escalated for approval.

  As requested in comment 6, we are complying with your journal’s policies about data sharing and are providing excel tables in the supporting information with these data. These files will include the cell counts from image files that were obtained in our experiments.

8. Please amend your list of authors on the manuscript to ensure that each author is linked to an affiliation. Authors’ affiliations should reflect the institution where the work was done (if authors moved subsequently, you can also list the new affiliation stating “current affiliation:….” as necessary).

 We have modified the title page to bring our manuscript in alignment with your publishing policies.

9. We note that you have included the phrase “data not shown” in your manuscript. Unfortunately, this does not meet our data sharing requirements. PLOS does not permit references to inaccessible data. We require that authors provide all relevant data within the paper, Supporting Information files, or in an acceptable, public repository. Please add a citation to support this phrase or upload the data that corresponds with these findings to a stable repository (such as Figshare or Dryad) and provide and URLs, DOIs, or accession numbers that may be used to access these data. Or, if the data are not a core part of the research being presented in your study, we ask that you remove the phrase that refers to these data.

 We address this concern in questions 6 and 7.

10. [Please include captions for your Supporting Information files at the end of your manuscript, and update any in-text citations to match accordingly. Please see our Supporting Information guidelines for more information: http://journals.plos.org/plosone/s/supporting-information.](https://nam12.safelinks.protection.outlook.com/?url=http%3A%2F%2Ftrack.editorialmanager.com%2FCL0%2Fhttp%3A%252F%252Fjournals.plos.org%252Fplosone%252Fs%252Fsupporting-information.%2F1%2F010f0196873c5369-494753d7-fe34-46c3-b215-91735291dfb5-000000%2FJbr0Rx3zpVNAE1ris9-p3jCGzbavpnrv0hYMZoLJPdY%3D208&data=05%7C02%7C%7C45171c4bce9c4de2fccd08dd87f8e7a3%7C31d7e2a5bdd8414e9e97bea998ebdfe1%7C0%7C0%7C638816225327487703%7CUnknown%7CTWFpbGZsb3d8eyJFbXB0eU1hcGkiOnRydWUsIlYiOiIwLjAuMDAwMCIsIlAiOiJXaW4zMiIsIkFOIjoiTWFpbCIsIldUIjoyfQ%3D%3D%7C0%7C%7C%7C&sdata=Pvl1LLfSyyheUSvv2D9OMSTuH76YLyrzhJDxMiOIXbk%3D&reserved=0)

 The captions for supporting information files are now located at the end of the manuscript.

[Note: HTML markup is below. Please do not edit.]

Reviewers' comments:

Reviewer's Responses to Questions

**Comments to the Author**

1. Is the manuscript technically sound, and do the data support the conclusions?

The manuscript must describe a technically sound piece of scientific research with data that supports the conclusions. Experiments must have been conducted rigorously, with appropriate controls, replication, and sample sizes. The conclusions must be drawn appropriately based on the data presented.

Reviewer #1: Yes

Reviewer #2: Partly

2. Has the statistical analysis been performed appropriately and rigorously?

Reviewer #1: Yes

Reviewer #2: Yes

3. Have the authors made all data underlying the findings in their manuscript fully available?

The [PLOS Data policy](https://nam12.safelinks.protection.outlook.com/?url=http%3A%2F%2Ftrack.editorialmanager.com%2FCL0%2Fhttp%3A%252F%252Fwww.plosone.org%252Fstatic%252Fpolicies.action%2523sharing%2F1%2F010f0196873c5369-494753d7-fe34-46c3-b215-91735291dfb5-000000%2FRqDlv0xS0iHahYyaLuQHn8zjoUgPpFwOWxp4OAW5RaA%3D208&data=05%7C02%7C%7C45171c4bce9c4de2fccd08dd87f8e7a3%7C31d7e2a5bdd8414e9e97bea998ebdfe1%7C0%7C0%7C638816225327518095%7CUnknown%7CTWFpbGZsb3d8eyJFbXB0eU1hcGkiOnRydWUsIlYiOiIwLjAuMDAwMCIsIlAiOiJXaW4zMiIsIkFOIjoiTWFpbCIsIldUIjoyfQ%3D%3D%7C0%7C%7C%7C&sdata=VenSKaSz%2BImJTW%2BzR47wOJ%2BvV%2B0%2Fqdj6dnpbYMK%2F7GY%3D&reserved=0) requires authors to make all data underlying the findings described in their manuscript fully available without restriction, with rare exception (please refer to the Data Availability Statement in the manuscript PDF file). The data should be provided as part of the manuscript or its supporting information, or deposited to a public repository. For example, in addition to summary statistics, the data points behind means, medians and variance measures should be available. If there are restrictions on publicly sharing data—e.g. participant privacy or use of data from a third party—those must be specified.

Reviewer #1: No

Reviewer #2: Yes

4. Is the manuscript presented in an intelligible fashion and written in standard English?

PLOS ONE does not copyedit accepted manuscripts, so the language in submitted articles must be clear, correct, and unambiguous. Any typographical or grammatical errors should be corrected at revision, so please note any specific errors here.

Reviewer #1: Yes

Reviewer #2: Yes

5. Review Comments to the Author

Please use the space provided to explain your answers to the questions above. You may also include additional comments for the author, including concerns about dual publication, research ethics, or publication ethics. (Please upload your review as an attachment if it exceeds 20,000 characters)

Reviewer #1: The general quality of the data presented in the manuscript is good, and the study addresses a relevant scientific question. However, the presentation of the data lacks clarity and, at times, appears sloppy. This significantly affects the readability and interpretation of the results. I have several major comments that need to be addressed to improve the manuscript.

Please see the attached review for more details.

Attached Review:

The general quality of the data presented in the manuscript is good, and the study

addresses a relevant scientific question. However, the presentation of the data lacks

clarity and, at times, appears sloppy. This significantly affects the readability and

interpretation of the results. I have several major comments that need to be addressed to

improve the manuscript.

1) Methods – Molecular Biology

"In vitro assembly (IVA)" – According to the original paper describing the method (https://

doi.org/10.1038/srep27459), IVA stands for ***In Vivo Assembly***, not ***In Vitro Assembly***.

 We thank the reviewer for pointing out this mistake, and we have corrected the methods section accordingly.

2) Results – “Capsaicin sensitivity of M308 mutants points to side chain constraints in

TRPV1 ARD”

Please describe the functional effects observed with the M308I substitution in more

detail.

We agree that this aspect of our findings needs to be more clearly described, and we have modified the results description accordingly,

“

Alanine and valine substitutions could not be distinguished from wild type in their capsaicin sensitivity measured by patch-clamp while the isoleucine substitution *at M308* incurred a *significant increase in sensitivity* to capsaicin. Therefore, our findings suggest that the fold between ankyrin repeats 4-5 imposes a unique space constraint on the side chain at position 308. Channels with an Ala, Val, or Met side-chain at position 308 behave just like WT, but insertion of hydrophobic residues Ile and Trp that are larger than methionine^19^ have a strong effect on TRPV1 channel function*, respectively increasing the channel’s sensitivity to stimuli or completely compromising function.*

“

3) Protonation of M308H modulates TRPV1 function

“The size-dependence of side-chain substitutions at M308, as well as a gain-of-function

phenotype observed when ***Ile is substituted for Met or Val*,** led us to hypothesize that

covalent modification of the side-chain could result in channel activation or sensitization.”

Did you mean substitution of Met to Ile or Val? Please clarify the direction of the mutation.

We thank the reviewer for catching this unclear passage, and we amended the statement to read:

“

*We observed that substitutions at M308 with side chains smaller than methionine retained WT channel function, whereas substitution with the slightly larger, and branched isoleucine increased channel activity in response to capsaicin, which leads us to hypothesize that covalent modification of the M308 side-chain could result in channel activation or sensitization*.

”

I also find the conclusions drawn from these experiments problematic. The data suggest

that M308H reduces expression or trafficking of functional channels to the membrane.

This could potentially be tested experimentally, but as it stands, the mutation appears

detrimental.

We thank the reviewer for their comment about the trafficking and expression of TRPV1-M308H to the plasma membrane. Our primary intent with the M308H substitution was to position a pH-sensitive residue in the protein fold that could tune channel function by altering the electrostatic environment at position 308, analogous to what would happen if the side-chain were covalently modified. Our conclusions about the altered pH sensitivity between WT TRPV1 and M308H are drawn from this paradigm. We agree that in addition to the altered pH-sensitivity in the TRPV1-M308H channel relative to WT there appears to be altered expression in the mutant. Nevertheless, a study of channel trafficking and expression levels in the mutant goes beyond our interest in addressing the questions we had about whether even small non-covalent alterations involving the side-chain at position 308 can modify channel activity.

Additionally, I am not an expert in single-channel recordings and analysis, but the data

presentation is not sufficiently clear. It would be helpful to include wild-type TRPV1 singlechannel

recordings and a comparison of open probability values.

We thank the reviewer for their suggestion to include single channel traces of WT TRPV1. Although there are no single channel records in our Figure 3 WT sample, we did have an excised patch with 5 WT channels where individual channel transitions are clearly detectable, and we also have one multi-channel patch (5 channels) experiment with M308H that was not included in the previous submission. These results are now included in Figure S5 to provide reassurance that our observations in multi-channel patches are the same as in single-channel patches for WT or M308H mutants. Both experiments follow the expected patterns of pH-dependence for the respective channel type previously demonstrated in Figure 3. Included in the text:

*“*

*Although most of the data collected for M308H was of single channels, we collected one complete recording of a multi-channel patch expressing M308H, which had a pH-dependent response profile consistent with what we observed in single channel patches (Figure S5).*

*”*

4) “A physiologically relevant role for this shorter splice variant could provide an

explanation for why the methionine at position 308 is so highly conserved, ***even though***

***other residues at that position result in channels with behavior that is similar to***

***WT.*”**

This is a controversial statement. You only demonstrated similar behavior for Ala, Val, and

slightly changed for Ile. The substitutions to Trp rendered the channel inactive. The His

substitution significantly reduced current amplitude.

We thank the reviewer for pointing out this apparent inconsistency, as the previous version of the text did not communicate our thinking effectively. We have now re-written this sentence to clearly point out that although other amino acids at position 308 result in similar channel behavior as methionine under the conditions that we tested, and can occur as missense variants in healthy individuals, the methionine at that position remains conserved between animal species and even other TRPV channel genes that are homologous to TRPV1. We therefore speculate that the predominance of the methionine at position 308 across TRPV1 orthologues and homologues could be related to its ability to function as an alternative start codon, which would give rise to a shorter variant with potential physiological relevance.

“

A physiologically relevant role for this shorter splice variant could provide an explanation for why the methionine at position 308 is so highly conserved *across species even though other amino acids at this position (ie. Ala, Val, Cys – see Figure S2) result in a channel with no or subtle changes to its function under the experimental conditions that we explored.*

“

5) Methods – Cell response counts from truncated TRPV1 co-expressed with TRPV1 wild

type

“Truncated constructs (TRPV1-preDelM308 and TRPV1-5’sv) were co-expressed with

TRPV1 wild-type constructs at a 25% to 75% plasmid DNA weight ratio: TRPV1 wild type

(25%) and PLC1-PH-SNAPtag (75%, a plasmid membrane marker for PIP2); TRPV1 wild

type (25%) and TRPV1-preDelM308 (75%); TRPV1 wild type (25%) and TRPV1-5’sv

(75%).”

It might be informative to try additional plasmid DNA ratios (e.g., 50:50 or 75:25). This

could help clarify how dominant the effect is. How many subunits of the truncated variant

are needed to prevent trafficking of the full channel to the membrane?

We thank the reviewer for their recommendation on an additional experiment. A pilot study was conducted to identify a ratio between WT TRPV1 and the truncated constructs to observe the dominant negative effect, and we landed on 25:75 as optimal. We planned our Figure 4 experiment around this ratio, with a control condition that relied on a plasmid containing plc-ph protein substituting for the TRPV1-PreDel DNA construct. We think it would be interesting to pursue research in the direction of how this mechanism operates, but we also find that this would extend beyond the current scope of study. In response to the second question, we find that panel E of Figure 4 sheds at least a small amount of light on this since no surface labeling of the TRPV1Δ1-308-exCellHalo was observed in the TRPV1 cotransfected cells. If a channel with at least one truncated subunit could be trafficked to the plasma membrane, then some signal from Alexa488 fluorescence would probably have been observed in this experiment.

**Minor comments**

6) Figure 1D

Correct "Argenin" to Arginine.

Done.

I recommend using the format MXXX rather than writing "Met in TRPA1 ARD" for

consistency and clarity.

The reference in panel D of Figure 1 is to all methionine positions in the ARD of TRPA1, which is described in the figure legend. We have modified the legend for clarification:

“

Relative abundance of missense variants in gnomAD *for all* methionine (Met) positions in ARD of TRPA1 (1-530) and *at M308 of* TRPV1. The most common amino acid encoded by missense variants in the 16 Met residues of *the ARD in* human TRPA1 is isoleucine (total of 33).

“

Consider using EC₅₀ instead of K₁/₂ to improve accessibility for a broader journal

audience.

We thank the reviewer for their suggestion and have made the recommended changes in the figures and text.

7) Figure 2C

Use °C instead of "deg C".

Also, use “WT” instead of “Wild type TRPV1” for consistency with other figures.

Done.

8) Figure 3B

In the zoomed-in regions of the current recordings (i.–iii.), the time markers do not appear

to match those in the main figure. Please double-check.

We thank the reviewer for catching this oversight and have corrected the figure.

Figure 3D

Use either Caps or Cap consistently throughout the figure and text when referring to

Capsaicin

Done

Reviewer #2: In the manuscript “Missense variant analysis in the TRPV1 ARD reveals the unexpected functional significance of a methionine.”, by Grace C. Wulffraat, Sanjana Mamathasateesh, Rose Hudson1, Benjamin He, Andrés Jara-Oseguera1, Eric N. Senning. Authors provide new data about isolates residue M308 in the ankyrin repeat domain of TRPV1 channel, which exhibits a large number of missense variants in humans while being conserved across species. Mutagenesis experiments have shown that substitutions in M308 can affect channel function, with larger or more polar residues increasing activity. In addition, the conservation of M308 may be related to its role in redox-dependent modifications, and a splice variant lacking M308 reduces TRPV1 expression on the surface of HEK293 cells. These data suggest that M308 is critical for channel regulation and expression dynamics.
Although the study is of undoubted interest and expands our knowledge about functions of TRPV receptors to increase the level of validity of the material and publication in the journal, it needs some improvements.
In the Materials and Methods section, the missense variant analysis, then electrophysiology. I would put the information about the object of study (Cell Culture) at the beginning. The electrophysiology section goes on to cell culture and it's not clear what kind of culture it is.

We thank the reviewer for their recommendation on placing the cell culture section before electrophysiology. We have reordered the sections on cell culture and molecular biology to appear before electrophysiology.

All the Materials and Methods sections are quite detailed, but the molecular biology section contains mostly references to literature, it should be expanded to include information on the experimental approaches used in the study.

We thank the reviewer for their comment about our molecular biology methods. The mutagenesis methods are standard across many labs and rather than repeating a rough scheme of the steps as was done in, Mott et al. (2023), we opted to cite those methods here as well as the original IVA protocol on which these techniques are based. We have, however, provided a spreadsheet with details about the primers used to generate the different mutants in this study.

Capsaicin was diluted in ethanol. Potentially ethanol can have an effect on cells . It is necessary to specify what concentration of solvent was used and whether the same concentration of solvent was added to the control solutions. If not, explain why this can be neglected.

Both DMSO or ethanol are commonly used to dissolve capsaicin in stock solutions which are further diluted into the working buffer solutions. We maintained final dilutions that were less than 1:1000 (v/v) EtOH to water, which is below the range of potential TRPV1 activation observed for ethanol (See Trevisani et al. (2002), Nat Neuro.). Moreover, the same capsaicin solutions and dilutions of ethanol were in experiments with wild type and mutant channels, and for WT channels we obtained responses that are fully consistent with previous measurements of the capsaicin sensitivity of TRPV1.

In the experiments with temperature change a modified setup was used, it is necessary to indicate how fast and with what accuracy it was possible to change the temperature in the experiment.

All modifications to the temperature-control system were made by the manufacturer to allow for faster changes in temperature without having an impact on the accuracy with which temperature is detected. This is because we used a thermistor bead located <5mm from the pipette tip at the same height from the chamber bottom to measure temperature, which is independent of the circuit controlling the temperature in the chamber. The specifications of the delta-T chamber indicate a <0.2 °C temperature differential across a large confinement zone where our pipette tip and thermistor sensor were placed in our experiments (https://bioptechs.com/delta-t-dish-incubation-system/). We started each experiment noting that the thermistor bead reported room temperature in the newly exchanged buffer solution.

The Materials and Methods section lacks information about the method of staining of the preparation in Fig. 4 E and the microscopic technique with which it was obtained. There is no scale in Fig. 4 E.

We thank the reviewer for their comment. We increased the size of the scale bar in panel E to improve its visibility and have modified the methods section to include more details about staining in Figure 4E:

“

*HEK293T cells were transfected with 50:50 DNA ratios of TRPV1 WT:TRPV1-exCellHalo in a control sample and TRPV1WT:TRPV1Δ1-308-exCellHalo in a test sample. TRPV1Δ1-308-exCellHalo was also transfected in isolation. Cells were stained after 24 hours with the Promega dyes Alexa488-HaloTag and JF549HaloTag, which are respectively cell impermeable and cell permeable. Coverslips were incubated in complete media with 1 μM of each dye type for 30 minutes at 37 degrees C before the media was exchanged and the cells recovered at room temperature for 10 minutes, followed by immediate imaging.*

“

There are two large sections in the Materials and Methods are devoted сalcium imaging experiments. But in the Results and Discussion section I found very little information regarding the description of these experiments. It is necessary to clearly describe why they were performed and what the authors wanted to show. Why ionomycin was used, what it allows to reveal, what follows from the obtained data. I did not find in the discussion section any specific conclusions on this block of results.

We thank the reviewer for bringing attention to this omission. We have modified the methods to more clearly explain our cell counting approach to characterizing the dominant negative effect of the truncated TRPV1 mutants and have included a passage in the conclusion section that highlights the results from the calcium imaging experiments:

“

We reasoned that testing channel activity with capsaicin alone was not sufficient to characterize TRPV1 functional properties pertaining to pathogenicity, or the pathogenicity prediction by Alphamissense was inaccurate. *Turning to our experiments that explore the dominant negative potential of shortened TRPV1 constructs revealed a significant drop in cellular responses to capsaicin when wild type TRPV1 is contransfected with the 5’sv construct.*

“

The same applies to data obtained by electrophysiological methods, and there is no discussion of these data or conclusions from these experiments in the discussion section.
I think it is also good to add to the discussion information about the physiological significance of the obtained results

We thank the reviewer for their recommendations and have attempted to tighten up the language of the conclusion section to improve readability.

With all the suggested additions, the paper can certainly be accepted for publication.

6. PLOS authors have the option to publish the peer review history of their article ([what does this mean?](https://nam12.safelinks.protection.outlook.com/?url=https%3A%2F%2Ftrack.editorialmanager.com%2FCL0%2Fhttps%3A%252F%252Fjournals.plos.org%252Fplosone%252Fs%252Feditorial-and-peer-review-process%2523loc-peer-review-history%2F1%2F010f0196873c5369-494753d7-fe34-46c3-b215-91735291dfb5-000000%2F2bh7akLeO6wyXbcATNCZuWAslOfKoYpjbO0750pumWA%3D208&data=05%7C02%7C%7C45171c4bce9c4de2fccd08dd87f8e7a3%7C31d7e2a5bdd8414e9e97bea998ebdfe1%7C0%7C0%7C638816225327544484%7CUnknown%7CTWFpbGZsb3d8eyJFbXB0eU1hcGkiOnRydWUsIlYiOiIwLjAuMDAwMCIsIlAiOiJXaW4zMiIsIkFOIjoiTWFpbCIsIldUIjoyfQ%3D%3D%7C0%7C%7C%7C&sdata=ZEWP79dUqRK8OqgOXfN0rkUjDJJkhwzuWeF0fmt8h6w%3D&reserved=0)). If published, this will include your full peer review and any attached files.


If you choose “no”, your identity will remain anonymous but your review may still be made public.


**Do you want your identity to be public for this peer review?** For information about this choice, including consent withdrawal, please see our [Privacy Policy](https://nam12.safelinks.protection.outlook.com/?url=https%3A%2F%2Ftrack.editorialmanager.com%2FCL0%2Fhttps%3A%252F%252Fwww.plos.org%252Fprivacy-policy%2F1%2F010f0196873c5369-494753d7-fe34-46c3-b215-91735291dfb5-000000%2Fht_qpeQD0u3BC5Jsephz1aSB2NnpC3wM6KajrDMRGDs%3D208&data=05%7C02%7C%7C45171c4bce9c4de2fccd08dd87f8e7a3%7C31d7e2a5bdd8414e9e97bea998ebdfe1%7C0%7C0%7C638816225327568077%7CUnknown%7CTWFpbGZsb3d8eyJFbXB0eU1hcGkiOnRydWUsIlYiOiIwLjAuMDAwMCIsIlAiOiJXaW4zMiIsIkFOIjoiTWFpbCIsIldUIjoyfQ%3D%3D%7C0%7C%7C%7C&sdata=eESmHixwARDeofln%2BSKMeaZx76V8XyppaEkB0qeMwlg%3D&reserved=0).

Reviewer #1: No

Reviewer #2: No
